# Supplementary material for: Are carbon emissions trading and green financial instruments synergistic? -Comprehensive quantitative research based on content analysis
Source: PLoS One. 2024 Mar 7;19(3):e0298601. doi: 10.1371/journal.pone.0298601 (PMC10919601; doi:10.1371/journal.pone.0298601)
Supplement: S1 Data — (ZIP) [file pone.0298601.s001.zip › Data/地级市Z_F工作报告.zip!/╜¡╬≈/─╧▓2╩╨/└┤╫╘╣1⁄2╓┌║┼í╛┬φ┐╦╩2╛▌═°í┐_2017.docx]

各位代表：

　　现在，我代表市人民政府向大会报告工作，请予审议，并请各位市政协委员和列席会议的同志提出意见。

　　一、2016年工作回顾

　　刚刚过去的2016年，面对错综复杂的国内外形势和艰巨繁重的改革发展稳定任务，在省委、省政府和市委的坚强领导下，在全市人民的共同努力下，我们主动认识、顺应和引领经济发展新常态，不断开拓进取、真抓实干，顶住了经济下行的巨大压力，各项工作取得了明显成效，全市经济社会呈现良好发展势头。

　　一是“十三五”实现开门红。2016年，各项主要经济指标保持了平稳较快增长，实现地区生产总值4354.99亿元，增长9.0%；财政总收入790.8亿元，同口径增长12.9%；地方一般公共预算收入完成402.2亿元，同口径增长11.6%；500万元以上固定资产投资4540.26亿元，增长13.5%；规模以上工业增加值1611.50亿元，增长9.2%；社会消费品零售总额1868.00亿元，增长11.8%；实际利用外资30.65亿美元，增长13.3%；城镇居民人均可支配收入34619元，增长8.4%，农村居民人均可支配收入14952元，增长9.2%；居民消费价格指数同比上涨2.1%。

　　二是首位度稳中有升。财政总收入、地方一般公共预算收入等指标增速全省排位均较2015年底前移1位（2015年增速分别列全省第2、第3位），财政总收入、地方一般公共预算收入、规模以上工业增加值、实际利用外资等4项指标占全省比重分别较2015年底提高1.0、0.7、0.7、0.1个百分点（2015年在全省占比分别为20.8、18.0、20.0、27.6）。

　　三是质量效益明显提升。收入结构持续改善，全市财政总收入、地方一般公共预算收入税收占比分别达到86.5%（省口径）和79.2%，均排名全省第一，分别高于全省平均水平8.1和10.8个百分点。产业结构持续优化，三次产业结构比例调整为4.2∶53.0∶42.8。第三产业对经济增长贡献率为47.7%，拉动经济增长4.3个百分点。高新技术产业增加值占规模以上工业增加值的比重为27.9%。规模以上工业全员劳动生产率达到34.43万元/人，工业经济综合效益指数为330.2%；万元地区生产总值能耗下降2%。

　　一年来，我们主要做了以下工作：

　　1.着力优平台强集群，产业层级加快提升。

　　一是现代制造业蓬勃发展。九大重点产业累计完成规模以上工业增加值1357.13亿元，同比增长9.1%；完成主营业务收入4755.81亿元，同比增长9.6%。其中，电子信息产业实现快速增长，食品、汽车等产业总量持续攀升，汽车产业突破800亿元。江铃集团成功拿到全国第七张纯电动汽车生产资质，开启了南昌新能源汽车新纪元；引进了华夏九州通航总部及运营基地、鸿鹄航空AC500等通用机型研发及制造、南方云天无人机制造3个航空产业项目，航空产业向全产业链延伸。南昌“光谷”建设成绩喜人，南昌LED产业创新示范园开工建设，市光电产业研究院和国家级检测中心筹备工作顺利推进。欧菲光2号产业园建设进展顺利，联思光电、鸿利光电建成投产，业际光电、东海蓝玉等项目密集开工建设，光电产业累计完成主营业务收入500亿元，同比增长30%。预计全市工业园区实现主营业务收入4800亿元，其中高新区1900亿元、经开区1110亿元、小蓝经开区1100亿元。县区产业平台不断提升，南昌县“一区带三园”产业升级模式全面推进，新建区长堎工业园入选长江经济带国家级转型升级示范开发区，进贤县经济技术开发区、临空经济区电子信息产业园获批省级产业园。

　　二是现代服务业快速增长。VR产业基地创新孵化中心正式启用，50余家企业签约入驻，VR产业开始起步。全省金融商务区升级步伐加快，落户各类金融企业215家。胜利路步行街、新洪城大市场项目加速推进，绳金塔商业街被评为全国特色商业街。万达茂及主题乐园项目开业，南昌绿地国际博览中心正式投入运营。万寿宫历史文化街区项目加快建设，滕王阁景区通过5A级景区景观质量评审。物流业加快发展，南昌市获评为全国物流标准化试点城市。全市旅游接待总人数和旅游综合收入快速增长，分别达到8301.1万人次、816.8亿元，增长50%、51.8%。“慧谷”建设稳步推进，九次方大数据公司正式落户，百亿“中科心客众创基金”正式启动，中航长江设计师创意产业园受到李克强总理充分肯定，获赞“创意无限、市场无限”。

　　三是现代农业加速推进。粮食生产总体平稳，实现建国以来首次“十三连丰”。农业主体不断壮大，国家级龙头企业12家，省级龙头企业111家，分别占全省的1/3和1/7，发展各类农业专业大户7846家，家庭农场8349家，农村合作社4330家，分别占全省的1/5、1/3、1/4。安义县江西绿能公司实行的“公司+生产大队+种养合作社+家庭农场”经营模式，受到习近平总书记的充分肯定。“绿谷”建设有声有色，“两区一廊”示范工程强力推进，溪霞店前古驿风情村、罗亭岭南电商产业村等10个特色秀美示范村建设基本完成。溪霞、罗亭、万埠、石鼻4个特色小镇改造提升效果明显，怪石岭农业休闲观光长廊、南昌菜园等一批都市现代农业观光休闲景区建设顺利推进。

　　2.着力抓改革促开放，发展动力明显增强。

　　一是改革红利加快释放。制定实施了《关于加快推进供给侧结构性改革的实施意见》、《南昌市关于大力促进实体经济发展的若干措施》等政策。企业事中事后改革进展顺利，全市企业监管大数据中心全面建成。深化行政审批制度改革，持续开展项目涉审中介机构诚信管控平台建设工作。积极创新重点产业投融资体制机制，大力发展多元结构的产业引导基金，PPP模式融资改革试点加速推进。国资国企改革和“营改增”改革深入推进。

　　二是创新创业动力强劲。出台了《关于深入推进创新驱动发展战略加快创新型城市建设的实施意见》，基本形成了“1+N”的科技创新政策体系。资金保障力度加大，全市财政科学技术支出10.17亿元，增长25.56%。成功入围“国家第二批促进科技和金融结合试点城市”、“首批国家重点产业知识产权运营服务试点城市”。“璀璨计划”技术创新与研发成果转化基地、国家重点实验室、博士后工作站加快建设，与中科院、同济大学、天津大学技术创新合作加快推进。全年专利授权8592件，增长36.9%，占全省总量27.3%。

　　三是内联外融纵深推进。省内融合稳步实施。昌九一体化有新的突破，实现了住房公积金贷款一体化，两市户口迁移实现“一站式”办理；昌抚一体化有序推进，“1+8”系列合作协议初见成效。省外协作加快开展。承办了长江中游城市群省会城市第四届会商会，共同签署了《南昌行动》；举办了第十二届泛珠三角区域省会城市市长联席会，加入了中英地方合作联合工作组、中德工业城市联盟；南昌至洛杉矶航线首飞，实现了全省洲际航线零的突破。

　　3.着力抓统筹提水平，城市品位提档升级。

　　一是城市容貌更靓。城市规划更加完善。《〈南昌市城市总体规划（2016—2030）〉编制工作方案》、《南昌市城市公共空间专项规划》、《南昌市综合管廊规划》、《南昌市燃气专项规划》等专项规划编制完成，启动了“多规合一”工作。建设力度不断加大。昌九高架一期工程、昌九大道、七里岗互通立交、南京东路道路改造和九洲高架全线通车。轨道交通2号线、3号线加快建设，4号线已经进入招投标阶段。棚户区改造投入110亿元，完成省里下达城市棚户区改造开工任务2.6万户。配套功能更加完善。南昌外国语学校新高中部已完成建设，即将投入使用。外国语学校初中部、九龙湖小学（南师附小）、腾龙学校、朝阳中学建设和南昌十二中扩建工程加快推进。省妇幼保健院红谷滩医院正在进行基础施工，省儿童医院红谷滩新院、省人民医院红谷新院主体施工已完成，正在进行室内外装修，南昌一附院象湖分院主体工程竣工封顶。停车场三年攻坚计划大力实施，41个停车场顺利完工，投入使用泊位数7935个。地下综合管廊建设有序推进。管理水平不断提升。环境卫生“马路本色”行动、“五车六乱”、“十二大专项”整治行动深入开展，“美丽南昌·幸福家园”综合整治全面启动，环卫保洁三级监督考评全覆盖监督机制已经建立，城市垃圾无害化处理率达到100%。

　　二是乡村面貌更美。26个“五位一体”综合示范村和46个中心村建设加快推进。全力打造18个特色小镇，进贤县文港镇被授予全国“特色小镇”称号。568个新农村点“七改三网”建设和通道“五化”整治成效明显。农村水环境治理试点工作顺利推进，乡村及农业企业污水处理站达到47个，梅岭、西山等8个乡镇的污水处理厂投入使用。村民建房管理进一步规范。全市农村生活垃圾无害化处理率达到了90%，高于全省平均水平。

　　三是生态环境更优。深入实施“蓝天、清流、净土”行动。“黄标车”加速淘汰，扬尘污染综合防治有序推进。“河湖长制”全面推开、成效显著，面源污染防治工作力度加大。全市空气质量优良天数达到318天，优良率为86.9%，在中部省会城市排名第一。集中式饮用水源地和赣江、抚河14个监测断面水质达标率均为100%。

　　4.着力增投入补短板，幸福指数持续攀升。

　　一是民生投入力度不断加大。全年共筹集资金约385亿元用于教育、医疗、社保、保障性住房、城乡社区事务等民生工程，筹集资金约220亿元用于28类510项惠民便民利民工程，切实解决了人民群众关心、关注的痛点、难点问题。

　　二是社会保障网全面织牢。就业规模不断扩大，城镇新增就业人数8.6万人，完成全年目标的125.2%；新增转移农村劳动力4.69万人，完成全年目标的141.1%。社会保障更加完善，五大保险超额完成，在全省率先启动机关事业单位养老保险基金运行工作，城乡居民医疗保险统筹力度加大。加强房地产调控，一手去库存，一手防过热，连续出台了《关于促进我市房地产市场持续平稳健康发展的若干意见》、《关于促进南昌市房地产市场持续平稳健康发展有关工作的通知》等政策文件，房价上涨得到明显遏制，从准热点城市逐步退出。打响了“产业、保障、安居”三大精准扶贫攻坚战，全市贫困人口由4.65万减少到2.4万，贫困村由80个减少到49个，贫困发生率由1.8%下降到0.95%，圆满完成了年度扶贫任务。

　　三是社会事业健康发展。医疗健康方面。县级公立医院综合改革深入推进，村卫生计生服务室标准化建设全面启动，全科医生（乡村医生）签约服务人数达170余万人，社区卫生服务中心业务用房国有化、固定化率达到100%，市直8家医院门诊“先诊疗后付费”流程改造全面完成。市第三医院朝阳院区、市第九医院重大疾病防控体系等一批重大项目加快推进。全面两孩政策稳步实施，计生服务水平进一步提高。市老年福利院等项目加快建设，全力打造了一批精品社区、精品居家养老服务点、精品敬老院，养老服务水平加快提升。教育方面。新增公办幼儿园34所，69所转办为普惠性民办幼儿园，新增学位1万余个，公办和普惠性民办幼儿园占比提高到82%。全市新增中小学用地面积620亩，新改扩建校舍20万平方米。全市民办学校发展到65所、在校生7.8万人。义务教育非政策性“零择校”成果进一步巩固。湾里、经开、红谷滩义务教育均衡发展通过国家专家组认定。文化体育方面。全市街道综合文化活动中心实现全覆盖，全面完成了有线电视“户户通”工程。南昌汉代海昏侯国遗址管理局挂牌成立，遗址保护利用工作有序推进。唱响城市名片，“天下英雄城”品牌深入人心。城市15分钟体育健身圈更加完善，2016年首届南昌国际马拉松、南昌市第十六届运动会成功举办。海军“南昌舰”退役返昌，军事主题公园项目加快推进，国防后备力量建设取得新的进步。

　　四是社会保持和谐稳定。积极开展信访积案化解攻坚和第三方参与信访工作，全力打造“阳光信访”、“责任信访”、“法治信访”。紧紧围绕“双提升”总目标，扎实开展打击“盗抢骗”等专项行动和综治工作“百日攻坚战”，全市发案数持续下降，破案率继续上升，公众安全感指数持续上升。全国综治“南昌会议”协助筹备工作获得充分肯定。积极推进律师制度改革，实现了市县乡三级法律顾问制度全覆盖。深入推进“六五”普法，荣获全国法治宣传教育先进城市。安全生产、消防安全、食品药品安全、道路交通安全、应急管理工作扎实推进，全市没有发生大规模群体性事件、重大治安案件、重特大火灾和安全生产事故。

　　五是政府自身建设得到加强。政府决策更加科学，行政行为全面规范。自觉接受各方监督，认真做好了人大代表建议和政协委员提案办理工作，媒体“啄木鸟”行动广受好评。12345政府服务热线平台顺利上线运行，政务公开力度加大。“两学一做”学习教育扎实开展，作风改进成效明显。“红包”、“小金库”和领导干部违规插手干预工程项目等问题专项整治深入开展，全市党政机关公务用车改革任务顺利完成，“三公”经费支出下降33.68%。

　　与此同时，统计、审计、物价、粮食、科普、人民防空、防灾减灾、民族宗教、外事侨务、对台事务、参事、史志、档案、保密、老龄、青少年、妇女儿童、爱国卫生、残疾人、消费者权益保护等工作，都取得喜人成绩。

　　各位代表，成绩来之不易。事实再次证明，有党中央、国务院，省委、省政府和市委的坚强领导，南昌人民有能力有信心战胜任何困难和挑战，不断夺取经济社会发展新的胜利!在此，我代表市人民政府，向全市人民，向所有关心、支持南昌发展的同志们、朋友们，表示衷心的感谢并致以崇高的敬意！回顾过去一年的工作，我们清醒地看到，经济社会发展还存在不少的问题和不足，主要是：经济总量不大，主导产业不强，创新能力不足，转型升级任务艰巨；制约发展的一些体制机制障碍依然存在，深化改革面临不少难啃的“硬骨头”；民生事业有不少短板，基本公共服务与人民群众期待还有差距；部分干部作风不硬，政府行政效能还有待提升，等等。对于这些问题，我们将以高度负责的态度，采取更加有效的措施，切实加以解决。

　　二、2017年主要工作

　　各位代表，2017年是实施“十三五”规划的重要之年，是供给侧结构性改革的深化之年，也是在全省率先全面建成小康社会的关键之年，做好政府各项工作至关重要。今年政府工作的总体要求是：全面贯彻党的十八大、十八届三中、四中、五中、六中全会和中央经济工作会议精神，深入贯彻习近平总书记系列重要讲话精神和治国理政新理念新思想新战略，特别是对江西工作的重要要求，认真落实省第十四次党代会、市第十一次党代会和市委十一届二次全会决策部署，紧紧围绕“打造核心增长极基本成型”目标，坚持稳中求进总基调，牢固树立和贯彻新发展理念，主动适应经济发展新常态，坚持推进供给侧结构性改革这一主线，全力主攻产业发展，加快打造城市品牌，不断强化辐射带动，持续提升民生福祉，全力保持经济社会持续健康较快发展，不断强化首位担当、展现省会作为，努力打造富裕美丽幸福江西的“南昌样板”，为把南昌建设成为工业文明、城市文明、生态文明融合发展的现代化城市奠定坚实基础。

　　今年经济社会发展的主要预期目标是：地区生产总值增长9%左右；财政总收入增长6%左右；地方一般公共预算收入同口径增长6%左右；全社会固定资产投资增长13.5%左右；规模以上工业增加值增长9%左右；社会消费品零售总额增长12%左右；实际利用外资增长10%左右；实际利用内资增长10%左右；出口总额与2016年持平；城镇居民人均可支配收入增长9%左右；农村居民人均可支配收入增长9.5%左右；居民消费价格总水平涨幅控制在3.5%左右。

　　今年政府将重点抓好以下几方面工作：

　　（一）围绕做强做大经济体量，突出抓聚焦强实力

　　一是坚持产业聚焦，进一步推转型促升级。

　　坚定不移推动工业转型升级。坚持新型工业化核心战略，以工业园区为主战场，围绕九大重点产业，注重谋篇布局，补链强基，集群发展，围绕产业链，推动技术链、人才链、政策链、资金链、平台链的深度融合。大力实施工业倍增计划。力争到2020年，工业主营业务收入突破11600亿元，高新区、经开区（含临空经济区）、小蓝经开区主营业务收入分别超过4000亿元、3000亿元和2000亿元，汽车及零部件产业、食品产业超过2000亿元，电子信息、生物医药、纺织服装、机电制造产业产值超过1000亿元。加快发展战略性新兴产业，力争到2019年，战略性新兴产业主营业务收入占全市工业总量比重提高10个百分点以上；战略性新兴产业增加值占全市工业增加值比重超过50%。大力发展电子信息产业，充分发挥高新区、经开区、临空经济区、青山湖区集聚效应，加快打造“南昌光谷”，将南昌打造成为全国有重要影响力的LED产业基地和移动通讯终端产业基地；大力发展航空制造产业，加快中航工业洪都商用飞机、瑶湖机场项目、通用航空制造及运营、无人机制造等相关产业项目建设；大力发展新能源汽车产业，加快实施保利协鑫新能源汽车生产基地、观致新能源汽车整车、江铃新能源汽车三个重大产业项目。大力发展生物医药产业，充分发挥济民可信、汇仁制药、江中制药等龙头企业的引领作用，着力引进一批生物医药重大项目，加快打造国家生物医药高技术产业基地。注重新旧融合发展，加快传统产业转型升级，不断巩固和提升纺织服装、机电制造、食品加工、新型建材等传统产业优势。着力完善工业功能平台。完善工业发展组织架构，健全产业发展推进机制。整合企业、高校等力量，成立产业发展研究院；加快建设高水平研发、检测、设计、展销、物流等公共服务平台；加快工业标准化厂房建设，力争2017年新建400万平方米；大力提升园区公建配套，加大供水、供电、供气、通信等强弱电管网建设。

　　坚定不移推动服务业转型升级。突出抓好生产性服务业。加快发展金融服务业，推动银行机构改善和创新服务，支持我市重点龙头企业建立资源要素交易市场。大力推进国家服务业综合改革试点建设，加快工业设计中心和工业设计基地申报工作。大力推动向塘铁路物流枢纽和陆路口岸、昌北多种交通方式衔接的物流枢纽建设，积极推进全国物流标准化试点项目建设，全面完成省级城市配送试点项目建设，稳步实施八大省级物流产业集群建设。加快打造“南昌慧谷”，积极推动中航长江设计师创意产业园、江西慧谷·红谷创意产业园、中国（南昌）虚拟现实VR产业基地、699文化创意产业园等产业项目建设，集中力量打造数字创意、文化创意产业集聚区。继续加快电子商务、总部楼宇等现代服务业发展步伐。突出抓好生活性服务业。加快红谷滩中央商务区和八一广场中山路市级商业中心建设，打造2至3条特色商业街；大力推进引导城乡居民扩大文化消费试点工作，加快南昌汉代海昏侯国遗址公园和华侨城大型综合文化旅游等项目建设，推进滕王阁、万达文化旅游城、万寿宫历史文化街区创建国家5A级景区和梅岭创建国家级旅游度假区；推出一批精品旅游线路，力争接待旅游总人次突破1亿，旅游总收入突破1000亿元。

　　坚定不移推动农业转型升级。调整农业产业结构。坚持“稳粮、优供、增效”，不断优化农业生产结构，提升农业发展效益。依托农产品物流等七大特色产业集群，加快建设现代农业加工产业体系。推动农业产业化加速发展。充分挖掘农业生态观光资源、立足农村传统文化特色，大力发展都市型休闲旅游农业。激活农业产业发展动力。深化农村产权制度改革，统筹推进农村土地征收、集体经营性建设用地入市、宅基地制度改革试点。充分利用“财政惠农信贷通”、农业产业化“转贷资金”，扶持龙头企业做大做强。持续大力培植农业种养大户、农民家庭农场、农民合作社等新兴经营组织。加快农业科技创新，积极实施“互联网+农业”行动，推动“123+N”的南昌智慧农业建设，大力培育一批电商村、淘宝村和涉农电商企业。持续推进“南昌绿谷”建设。推进24个市级以上都市现代农业示范园区优化提升。按照“一乡一园”的思路，大力推进各种适度规模的农业园区建设。大力推进农业招商引资，引进一批大企业、大集团落户“南昌绿谷”。加快梅湖景区花博园项目建设，确保省第四届花博会今年4月如期举行。

　　二是坚持区域聚焦，进一步优布局扩开放。

　　全面优化产业布局。鼓励各县、区依托原有基础、发挥自身优势，发展壮大各具特色的区域经济主导产业和产业集群，着力形成错位发展、相互促进的区域经济板块。

　　大力开展招商引资。充分利用赣港赣台、中英地方合作联合工作组、中德工业城市联盟、世界赣商大会、洪商大会等重要平台，创新招商理念和模式，开展精准招商，通过招大引强、增资扩股等，大力推动全产业链招商，力争引进一批有品牌、有品质的龙头企业，引导外部资金和先进技术更多投向高端制造业、现代服务业和都市农业。

　　构建完善开放平台。确保实现南昌综合保税区封关运行，加快打造南昌开放型经济新平台；运用向塘铁路枢纽、龙头港国际码头、昌北国际机场等功能平台，努力拓展铁路、公路、水运、航空互联互通的开放通道。积极推进中欧（南昌—鹿特丹）国际铁路货运班列和海铁联运健康发展。

　　着力优化开放体制。积极推进构建开放型经济新体制综合试点试验工作，主动对接、借鉴复制现有国家级自贸区试点经验，力争形成一批可复制、可引领、可推广的经验和模式。

　　全面加强区域合作。深入对接“一带一路”战略，积极融入长江经济带，深度融入长江中游城市群，着力加强与闽浙赣皖福州经济协作区、泛珠三角省会城市等协作区的经贸合作和高层互动；全力支持和配合赣江新区建设，推进昌九、昌抚在更高层次、更宽领域开展合作。加大“走出去”力度，组织引导龙头企业开拓“一带一路”沿线国家和地区工程市场，着力打响“南昌建设”品牌。

　　三是坚持资源聚焦，进一步强平台优配置。

　　将资金、土地、人才等要素资源，更加有效地配置到重点领域、重大项目上来。

　　集聚金融资源。持续做大金融平台，稳步推进全省金融商务区做大做强，加快建设江西金融发展研究院、江西互联网金融产业园和江西基金产业园，加快建设金融服务平台和金融控股平台，加快组建千亿发展基金。千方百计破解企业融资难题，推动符合条件的企业在主板、创业板、新三板上市、挂牌、融资，支持上市公司再融资、并购重组；支持企业发行企业债、公司债、中期票据、私募债等债券，不断扩大金融直接融资规模。

　　整合土地资源。科学编制年度土地供应计划，结合旧城、旧厂、旧村（城中村）“三旧”改造，加大征迁收储和规划调整等工作力度，加快“净地”出让进度；加强新增用地计划指标精细化管理，提高指标使用效益，继续加强闲置土地清理，盘活用好存量土地资源，全力保障重点领域、重大项目用地需求。

　　激活人力资源。创新人才引进理念，大力推动人才引进理念由单纯的政策留人向事业留人与政策留人“双轮驱动”转变，不断增强人才的向心力和凝聚力。创新人才引进模式，着力推进国家级人力资源服务产业园建设，助推人力资源产业化。充分发挥企业主体性优势，通过政府购买服务的方式，鼓励企业、中介、猎头公司市场化引进人才。深入实施“洪城特聘专家”、“洪城计划”、“海鸥计划”等重点人才引进培训计划，探索灵活高效的人才激励政策和人才公寓等精准化人才服务，着力推动人才服务从单一的“报酬化”向“个性化”和“精细化”转变，着力引进一批“高精尖缺”人才。继续实施“211”培训计划、首席技师培养计划和“洪城工匠”评选活动，提升本地人才水平，为我市产业发展提供经营管理和高级技能人才支撑。

　　四是坚持政策聚焦，进一步抓改革促创新。

　　突出提升改革成效。以促进实体经济发展的120条政策措施为具体抓手，全面推进供给侧结构性改革，持续推进“三去一降一补”，深入推进“放管服”改革，持续深化“三单一网”改革，加快流程再造，提高审批效能，推动政务服务网向乡镇（街道）、村（社区）延伸；坚持“房子是用来住的、不是用来炒的”的定位，加强房地产市场运行监测和供需双侧调控，建立健全房地产市场平稳健康发展的长效机制。进一步深化国资国企改革，稳妥开展职业经理人、混合所有制改革及员工持股等改革试点工作。大力推动中心镇扩权强镇改革。积极争取创建国家社会信用体系建设示范城市，加大失信惩戒力度，进一步增强全社会诚信意识，不断改善提升发展软环境。积极稳妥发展混合所有制经济，鼓励、支持、引导非公有制经济发展，支持非公企业做大做强。

　　全面激发“双创”活力。顺应新经济发展的时代潮流，大力实施创新驱动发展战略，集中力量和资源，加快开发新技术、发展新产业、创造新模式，不断催生经济发展新动能。优化“双创”服务。健全创业服务体系，为“双创”开辟“绿色通道”，扶持各类群体创新创业。通过“互联网+”，积极发展众创、众包、众扶、众筹等新模式。加大创新成果转化政策支持力度，提升科技保险和知识产权质押融资效益。以入围首批全国重点产业知识产权运营服务试点城市为契机，采取市场化运作模式，促进知识产权转化运用。加快科技创新。全力打造以南昌高新区为龙头的“1+4”鄱阳湖国家自主创新示范区，全面推进南昌（国家）大学科技城建设，加快科技创新步伐，突出企业科技创新和转化主体地位，积极打造以工程技术研究中心、重点实验室为代表的企业创新平台，着力推动企业协同创新，探索以项目带动创新的体制机制，力争新增国家级高新技术企业80家。强化资金保障。以成功入围国家第二批促进科技和金融结合试点城市为契机，充分发挥财政科技投入的引导作用，鼓励支持具备条件的商业银行设立科技支行，设立“南昌市科技信贷风险补偿资金池”，启动“洪城科贷通”业务。营造良好氛围。完善青年创业服务体系，继续开展青年创新创业大赛等各类创业活动。培育勇于探索的创业家精神和敢为人先的创新创业文化，在全市形成鼓励创新、宽松容错的浓厚氛围。

　　（二）围绕建设宜居宜业现代化城市，突出抓统筹创品牌

　　一是优化城市空间布局。加快总体规划修编，推进“多规合一”和南昌市总体城市设计。大力推进九龙湖新城、儒乐湖新城、瑶湖新城、望城新区建设，统筹推进洪都新城、朝阳新城建设，加快湾里融入主城区步伐。深入推进棚改旧改、城中村改造，对老城分片区进行改造，推进青山湖西岸、临江商务区、洪都文化创意区、坛子口服务业繁华区建设，规划建设一批城市交通枢纽型服务业集聚区。

　　二是完善城市综合功能。加强城市基础设施建设。切实加快城市“三环”及“十纵十横”快速路网、揽山入城路网等重大基础设施建设。全力推进洪都大道高架、昌南大道改造、昌九大道改造二期等项目，完成沿江南北大道快速路、红谷隧道、绕城高速公路南外环建设工程，力争开工建设西外环绕城高速公路、二七过江通道项目，形成“覆盖城区、环网结合、互联互通”的城市快速路网系统。加快推进轨道交通建设，力争2号线8月份首通段建成通车，完成3号线所有站点一期主体围护结构施工，4号线开工建设。 积极推进“海绵城市”、“地下综合管廊”建设和装配式建筑发展。优先发展城市、城际公共交通，加快推进“公交都市”创建工作，切实提高城市公共交通分担率。深入开展停车场建设三年攻坚计划，加快推进公共停车场建设。加快推进师大附中滨江分校二期、九龙湖小学、腾龙学校、红谷二中等项目建设。打造“小而全、小而美、小而绿（再生资源回收）”的社区商业服务平台。

　　三是加快城市品质提升。积极探索城市管理的长效机制，完善城市综合管理各项法制保障，提升城市综合管理水平，重点推进“美丽南昌·幸福家园”环境综合整治行动。大力实施市容环境整治工程。重点治理农贸市场、背街小巷等八大场所，整治摊点乱摆、垃圾乱扔等七种乱象，推进老旧社区改造、城市积水点改造等五大项目，全面提升环卫保洁水平，净化城市空间。大力实施城市交通畅通工程。积极稳妥推进南昌市出租汽车行业改革，开展超限超载车辆运输集中整治，加大对机动车、非机动车、行人等交通乱象整治力度，重点加强对非机动车的行驶管理，深入开展“五车六乱”违法专项整治，确保各项整治取得明显效果。大力实施城市文明提升工程。深入开展“三风”活动，不断加强文明引导，积极开展志愿服务等活动，努力营造未成年人健康成长的社会文化环境，全面推进协商民主，不断提升市民文明素质和城市文明程度。

　　四是加快新农村建设。按照“整洁美丽，和谐宜居”的目标，突出“精致建设、精美呈现、精细管理”的要求，坚持全域规划、沿线连片、建一片成一片的思路，2017年，着力推进2000个左右新农村村点的“七改三网”建设，建设一批中心村“8+4”公共服务项目，重点推进南昌绿谷“两区一廊”沿线以及贫困村新农村建设全覆盖，力争用三年时间实现新农村建设“扫一遍”。同时坚持“镇村园联动、农文旅结合”，着力打造建设一批特色小镇、“五位一体”综合示范村、新户型示范村，全面提升新农村建设整体水平。

　　五是大力彰显生态优势。倍加珍惜一流的生态环境，坚定不移地走绿色崛起之路。深入推进蓝天行动，大力推进工业废气治理、限燃区高污染燃料锅炉淘汰改造，全面完成年度化学需氧量、氨氮、二氧化硫、氮氧化物四项指标减排任务，努力保持空气质量优良率在中部省会城市排名第一。深入推进清流行动，全力推进“河湖长制”工作，加强河湖水域岸线管理，推进区域性水系连贯，切实加强饮用水水源地环境监管，加强工业企业水污染防治监管、农村饮用水源环境监管、污水处理厂建设与管理和鄱阳湖环境保护，大力开展沿湖沿线村镇生活污水、畜禽养殖污染、农业面源污染和工业污染整治，力争主要江河（赣江、抚河）断面水质达标率高于92%，城市集中式饮用水源地水质达标率达到100%。加大赣抚尾闾综合整治工程推进力度，续建赣东大堤风光带、梅湖景区花博园改造提升、乌沙河综合整治、抚河故道末端蓑衣荚水生态治理等工程建设。深入推进净土行动，大力推进土壤环境质量监测、土壤质量风险评价、新增土壤污染防控、重金属行业企业污染防治等工作，严格新建项目环保准入条件、严格执行环保“三同时”制度，避免新建项目对土壤造成污染。定期对涉重金属生产企业周边土壤例行监测，严密监控土壤环境质量。深化生态文明体制机制改革，严格遵守《南昌市生态保护红线区划》，建立生态保护红线监管平台，开展国家重点生态功能区县域生态环境质量监测与评估，实现生态保护红线以及环境容量红线、产业准入标准等动态监控与综合评估。

　　（三）围绕实现全面小康社会目标，突出惠民生增福祉

　　坚持为民服务宗旨，更加注重民生改善，更加注重公平正义，大力发展各项事业，大力推进民主法治，持续实施百项惠民便民利民工程，不断增强百姓的获得感和幸福感。

　　一是大力实施富民增收计划。进一步提高城乡低保标准，提高企业未参保退休职工人均养老生活补助标准、企业退休人员养老金和城镇居民基本医疗保险补助标准，努力提高我市一线职工特别是农民工及其他普通职工的工资水平。加快推进各领域工资制度改革，积极稳妥推进县以下机关建立公务员职务与职级并行制度，加快形成橄榄型分配格局，力争我市总体工资水平在全国省会城市进位，人才报酬全省第一，城乡居民人均可支配收入全省第一，城镇和农村居民人均可支配收入分别达到37730元和16370元。

　　二是深入实施就业优先战略。全面落实和完善就业创业政策，抓好特殊群体的就业工作，千方百计吸纳高校毕业生就业。深入实施就业精准扶贫计划，全面开展贫困人员就业情况调查，建立台账动态管理。大力开展公共就业和人才专项服务活动，加强区域一体化用工招聘求职平台建设，不断完善市场化合作培训机制。大力推进我市公共就业和人才服务机构改革以及劳动力市场整合工作，努力促进各类群体的高质量就业。

　　三是着力强化社会保障。建立健全统筹城乡、更加公平、可持续发展的社会保障制度，积极推进全市机关事业单位五险统征制度改革，启动建立机关事业单位职业年金制度。继续做好我市城乡居民医保统筹工作，稳步推进扩面征缴工作，创新经办管理体制机制，扩大医疗保险异地就医即时结算范围和区域。大力推进居家养老服务工作，推进“五个统一”的居家养老服务中心规范化建设。运用“互联网+”技术，推进“智慧养老”试点工作，让老年人足不出户就能享受养老服务。进一步加强社区养老服务平台建设，提升农村敬老院建设水平，积极推进医养融合发展，全年新增养老床位5000张以上。采取“公建民营”、“民办公助”、服务外包等形式，鼓励引导社会资本参与养老事业，构建多层次、多样化的养老服务供给体系。

　　四是确保完成脱贫攻坚。坚持把脱贫攻坚作为头等大事和最重要的政治任务，大力实施精准扶贫，全力推动光伏扶贫、保障扶贫、安居扶贫、结对帮扶、村庄环境整治、主要基础设施建设、公共服务等七个全覆盖，实施光伏扶贫54个村站和1760个户站建设，确保全市2.4万贫困人口全面脱贫、49个贫困村全部退出，实现率先在全省全面脱贫的目标。同时，健全脱贫长效管理机制，构筑精准脱贫大数据平台体系，积极建立第三方评估监督机制，建立健全结对长效帮扶机制，确保稳定脱贫不返贫。

　　五是加快发展各项社会事业。制定实施第三期学前教育行动计划，加大小区配套幼儿园回收转普力度，扩大普惠性幼儿园覆盖面；加快推进学校新改扩建及维修改造各类建设，坚持实行义务教育划片区入学管理，确保非政策性“零择校”；深入推进教育集团发展带动战略，推进城乡义务教育一体发展；加快普通高中教育特色发展，积极构建现代职业教育体系；切实加强特殊教育和民族教育；扎实推进社区教育和学习型城市建设；加快推进校长教师交流轮岗，积极探索教师编制管理和人事制度改革，确保我市教育综合实力全省领先。全面启动市级公立医院改革，建立现代医院管理制度；加快推进市第一医院九龙湖医院、老年病医院、市卫生职业学院新校园项目建设；健全农村医疗卫生服务网络和城市社区医疗卫生服务体系；提高突发公共卫生事件应急处置能力，全面开展全市慢性病检测工作，加快推进居民健康卡发行工作；大力开展爱国卫生运动，巩固国家卫生城市成果；积极引导社会资本进入医疗卫生领域，大力推进中医药健康服务业发展。深化计划生育登记服务制度改革，抓好优生优育政策落实，切实提升我市妇女儿童健康水平。大力弘扬人文关怀，加强对残疾人的关心关爱。进一步挖掘整合历史文化资源，唱响南昌文化品牌，不断繁荣发展文化事业，统筹谋划好“省三馆”（省科技馆、省博物馆、省图书馆）现有场馆的接收、改造提升和运营管理等。认真贯彻实施《南昌市全民健身实施计划（2016—2020）》，编制公共体育健身服务设施规划，积极推进场馆场地建设；着力办好2017南昌国际马拉松赛事。持续巩固全国双拥模范城“八连冠”的优秀成果，全力做好建军九十周年纪念活动，加快军事主题公园建设。

　　六是改进创新社会治理水平。加快推进市应急联动指挥平台（二期）、天网工程、数据警务、智能化手段等科技强警和基础项目建设，建立立体化、现代化治安防控体系。全力推进信访积案化解，加快信访信息化建设进程，创新“阳光信访”新模式，及时回应群众诉求。大力实施食品安全战略，深入开展食品药品安全专项整治和质量提升行动，完善食品安全治理体系，积极创建食品安全示范城市。加强河道采砂管理工作，确保河道采砂安全、稳定、科学、有序，保障省市重大重点工程用砂。全力推动“双提升”工作，继续加强安全生产管理和应急管理，持续推进“平安南昌”、“法治南昌”建设，全力维护社会和谐稳定，切实增强群众安全感和幸福感。广泛开展“兴家风、淳民风、正社风”活动，不断提升市民文明素养和城市文明程度。

　　新的目标、新的任务，对政府自身建设提出了新的更高要求。在新的一年里，我们将全面提升政府治理现代化水平。坚持依法行政。不断加强和改进政府立法制度建设，健全依法决策机制，抓好行政决策合法性审查和政府法律顾问工作，不断加强政府智库建设，提高决策科学化水平。强化规范性文件备案审查制度和能力建设，不断深化行政执法体制改革，严格规范公正文明执法，强化政府法制监督，自觉接受市人大及其常委会法律和工作监督、市政协民主监督，切实做好建议、提案办理工作，着力提高政府法治工作水平。坚持阳光理政。深入推进政务公开，加强政策解读，主动回应社会关切，完善在线访谈、网络问政、12345政府服务热线等公众参与平台，扩大“两微一端”等政务新媒体信息发布受众面，推动政务决策、执行、管理、服务和结果全过程公开，主动接受群众监督和媒体监督。坚持实干施政。大力发扬苦干实干作风，树立“实在、实绩、实干”导向，狠抓重大任务落实，突出重点项目落地。不断健全问责机制，优化完善绩效考核体系，以铁的纪律抓落实，做到真抓实干、为民担当，切实打通政策落实的“最后一公里”。坚持勤政廉政。加强机关作风建设，大力开展懒政怠政、为官不为整治活动，不断提高政府行政效能。坚持把纪律和规矩挺在前面，坚决贯彻党内政治生活准则和党内监督条例，认真落实中央八项规定，持之以恒正风肃纪，坚定不移反腐倡廉，倾力塑造风清气正的政府良好形象。

　　各位代表，新的征程已经开启，伟大事业催人奋进。让我们紧密团结在以习近平同志为核心的党中央周围，在省委、省政府和市委的坚强领导下，同心同德、开拓创新、积极作为，为打造富裕美丽幸福江西“南昌样板”而努力奋斗，以优异成绩迎接党的十九大胜利召开！
